# Association between energy drink intake, sleep, stress, and suicidality in Korean adolescents: energy drink use in isolation or in combination with junk food consumption

**DOI:** 10.1186/s12937-016-0204-7

**Published:** 2016-10-13

**Authors:** Subin Park, Yeeun Lee, Junghyun H. Lee

**Affiliations:** 1 Department of Research Planning, Mental Health Research Institute, National Center for Mental Health, Seoul, South Korea; 2 Department of psychology, Korea University, Seoul, South Korea; 3 Department of Psychiatry, National Center for Mental Health, 127, Yongmasan-ro, Gwangjin-gu, Seoul, 04933 South Korea

**Keywords:** Energy drinks, Junk food, Mood, Suicide, Adolescents

## Abstract

**Background:**

A considerable amount of research suggests that the frequent use of caffeinated energy drinks may be associated with undesirable effects, particularly so in children and adolescents. This study aimed to investigate the associations between energy drink intake and mental health problems, in isolation or in combination with junk food consumption, in a nationally representative sample of Korean adolescents.

**Methods:**

Data from the 2015 Korean Youth Risk Behavior Web-Based Survey, collected from 68,043 adolescents aged 12–18 years (mean age 15.09 ± 1.72 years), were analyzed. Questionnaires were administered to collect information related to dietary behavior including energy drink intake and junk food consumption. Single item measures of sleep dissatisfaction, stress, depression, suicidal ideation, suicide plan, and suicide attempt were also administered. Associations between energy drink intake and sleep dissatisfaction, perceived severe stress, persistent depressive mood, and suicidality were investigated, and a multivariate approach was taken so that additional variance from demographic and lifestyle factors could be controlled for statistically.

**Results:**

Energy drink intake was significantly associated with sleep dissatisfaction (adjusted odd ratios [AORs] = 1.64 and 1.25), severe stress (AORs = 2.23 and 1.38), depressive mood (AOR = 2.59 and 1.51), suicidal ideation (AORs = 3.14 and 1.43), suicide plan (AORs = 4.65 and 1.78), and suicide attempt (AORs = 6.79 and 1.91), with a higher risk for more frequent use of energy drinks (≥5 times/wk) than for less frequent use (1–4 times/wk). The detrimental effect of energy drinks on mental health was particularly prominent in frequent junk food consumers.

**Conclusions:**

Our data suggest that energy drink intake had detrimental effects related to stress, sleep dissatisfaction, mood, and suicidality, in isolation or in combination with junk food consumption, in Korean adolescents. However, the cross-sectional study design prevents our ability to assess causal relationships.

Energy drinks are highly caffeinated soft drinks that are marketed as providing mental and physical stimulation and improving energy, athletic performance, and concentration [1]. Concern has been raised because such products sometimes bypass legislation regarding caffeine content [2, 3], and therefore have the potential to put users at risk of intoxication [4, 5]. Energy drinks did not become widely available in South Korea until 2010. Energy drinks boasted an explosive total volume growth of 665 % from introduction in 2010 to 2012, when consumption totaled around 19.9 m liters [6]. Particularly, among teenagers who are overburdened with studying and examinations, the consumption rates are increasing because of its concentration enhancement and awakening and fatigue-relieving effects [5, 7]. Students in South Korea study longer hours and sleep shorter hours than students in other Organization for Economic Cooperation & Development (OECD) countries. In 2009, Korean students studied an average of 7 h 50 min per day, compared to 5 h 21 min in Japan, 5 h 04 min in the US, 3 h 49 min in England, and 6 h 6 min in Finland, and slept an average of 7 h 30 min per day, compared to 8 h 47 min in the US, 8 h 36 min in England, and 8 h 31 min in Finland [8].

Caffeine and sugar content are the main ingredients in energy drinks [9, 10]. Caffeine is most widely known as central nervous system stimulant, which activates noradrenaline and serotonin neurons [11]. Caffeinated energy drinks may also cause activation of methylxanthine [12, 13] which may related to psychological condition including memory, anxiety, or sleep [11], even though these effects seem to vary according to individual sensitivity to the methylxanthine [11, 13]. Excess sugar is also likely to linked to low level of serotonin and brain function [14]. Caffeine use does appear to have important implications in the school environment. Its high consumption has been associated with difficulty sleeping and tiredness in the mornings [15], falling asleep at school [16], behavioral problems [17], violence and conduct disorder [18], and low academic achievement [19].

The frequency and amount of consumption of many food and drink products is known to be highly inter-correlated [20]. For instance, fast food consumption is positively associated with energy drink intake and soft drink consumption, and negatively associated with fruit, vegetable, and milk intake in adolescents [21]. The effects of energy drinks may be best understood in combination with other aspects of diet. With increasing consumption of high-caffeinated energy drinks, an increase in the intake of junk food (processed and snack foods, which are high in fat and/or sugar) is of particular concern in Korean children and adolescents [22]. Previous studies found a relationship between a junk food diet and hyperactive behaviors, depressed mood, and lethargy in children and adolescents [20, 23, 24]. Thus, both energy drink and junk food consumption may adversely affect adolescents’ mood and behavior.

The current study aimed to examine the associations between energy drink intake and self-reported sleep dissatisfaction, stress, depression, and suicidality in a nationally representative sample of Korean adolescents. The combined effects of energy drink intake and junk food consumption were also investigated. It was hypothesized that 1) energy drink intake is related to mental health problems such as sleep dissatisfaction, depression, and suicidality and 2) frequent energy drink intake in combination with frequent junk food consumption would be the strongest predictor of such undesirable outcomes.

## Methods

### Participants and procedures

Data from the 2015 Korean Youth Risk Behavior Web-Based Survey (KYRBS) were used. The KYRBS is an anonymous self-reported online survey conducted annually since 2005 by the Korea Centers for Disease Control and Prevention to identify teenagers’ health behaviors with the subjects ranging from middle school freshmen to high school seniors. Using stratified cluster sampling, the middle and high school student samples that represent the teenager population were extracted. After given the instructions of the survey, the students who agreed to participate did the survey during class. Personal data including the respondents’ names, schools, phone numbers, addresses, and resident registration numbers were not collected. In 2015, the survey included a total of 800 schools, i.e. 400 middle schools and 400 high schools each, and a total of 70,632 respondents participated. Regarding gender, 35,204 male students and 32,839 female students participated, reaching 96.7 % of participation rate. The average age of the respondents was 15.09 ± 1.72, ranging from 12 to 18. As it can be searched from other sources easily [25], the description of sampling method and survey process will not be discussed in detail further. The KYRBS was reviewed and approved by the institutional review board of Korea Centers for Disease Control and Prevention (2014-06EXP-02-P-A). The KYRBS data used for the present study is openly available [26].

### Measurements

#### Predictor variables

Frequency of energy drink intake during the previous week was assessed on a seven-point scale where 1 = *never*, 2 = *once or twice a week*, 3 = *three or four times a week*, 4 = *five or six times a week*, 5 = *once a day*, 6 = *twice a day*, and 7 = *three times or more a day*. Participants were classified into three groups: highly frequent energy drink intake (≥5 times/week), moderately frequent energy drink intake (1–4 times/week), and infrequent energy drink intake (<1 time/week).

#### Covariates

Sociodemographic variables included gender, age, school type (middle school, high school), place of residence (name of city), and residential type (residence with family, with relatives, with friends, alone, dormitory, or residence in a facility). Places of residence were dichotomized as rural vs. urban area, and residential type was dichotomized as residence with vs. without family. Perceived academic achievement was assessed using a 5-point Likert scale (high, high-middle, middle, low, low-middle, and low) and further dichotomized as high achievement vs. others.

The frequency of intense-level physical activities was evaluated asking the following. “In recent 7 days, how many days did you engage in physical activities (such as, riding bikes at a rapid speed, lifting up heavy loads, swimming fast and so on) for more than 20 minutes that made you sweat or pant heavily?” The scales were given from 1(none) to 6(more than 5 days a week). Those who engaged in moderate level physical activities for more than five times a week or those who engaged in intense level physical activities for more than three times a week were categorized as ‘physically active’ group [10, 27].

Frequency of junk food consumption during a previous week was assessed on a seven-point scale where 1 = *never*, 2 = *once or twice a week*, 3 = *three or four times a week*, 4 = *five or six times a week*, 5 = *once a day*, 6 = *twice a day*, and 7 = *three times or more a day*. Junk food consumption was dichotomized as infrequent consumption (≤2 times/week) vs. frequent consumption (≥3 times/week). Lifetime alcohol use was assessed with the following question: “Have you ever used alcohol?” The response options were “yes” or “no.”

#### Outcome variables

The degree of sleep dissatisfaction was measured with the following question: “In the last week, how satisfactory was your sleep in terms of relieving your fatigue?” The response options were very satisfactory (1), satisfactory (2), average (3), unsatisfactory (4), and very unsatisfactory (5). On the basis of the responses, participants were classified into the following two groups for multivariate logistic regression analyses: ≥ average satisfaction with sleep (1–3) and (ii) < average satisfaction with sleep (4–5) [27].

The degree of perceived stress was measured as following: “Usually, to what degree are you stressed? The answers ranged from 1) not at all 2) not so much 3) a little bit 4) quite much 5) very much [26].

The depressed feeling experience in the recent 12 months was measured as following: “Have you experienced sadness or despair to the degree that stopped your daily routine for two weeks?” [26].

Suicidal ideation was evaluated with the following question: “In recent 12 months, have you ever thought of committing a suicide?” Suicide plan was evaluated with the following question: “In recent 12 months, have you planned a suicide in detail?” Suicide attempt was evaluated with the following question: “In recent 12 months, have you ever attempted a suicide?” [26].

#### Statistical analyses

Univariate logistic regression tests were performed to compare sociodemographic and lifestyle factors between groups (high energy drink use vs. non-use; moderate energy drink use vs. non-use). Odds ratios (ORs) and 95 % confidence intervals (CIs) were calculated using energy drink intake (highly or moderately frequent intake) as the predictor and each sociodemographic and lifestyle factor as the main outcome variables.

Next, to analyze the associations between the adolescents’ energy drink intake and mental health variables, multivariate logistic regression tests were performed using energy drink intake as the principal predictor and each mental health variable as the main outcome variables, after controlling for age, gender, school type, area of residence, residential type, level of academic achievement, level of physical activity, and junk food and alcohol consumption.

Next, in order to further examine the nature of the relationships observed, the variables for frequency of energy drink and junk food consumption were combined so that all four possible groupings of frequent/infrequent intake could be investigated in relation to the dichotomous mental health outcomes, using binary logistic regression analysis. Because consuming energy drinks less than once a week and junk food less than three times a week were considered to be the healthiest dietary practices, the infrequent junk food/energy drink condition was chosen as the comparison group. If the confidence intervals were found not to overlap, then the difference between groups was considered to be significant.

All analyses took into account the sampling design parameters, weighting, clustering, and stratification factor. The proportion of general subject characteristics was weighted according to the respondent’s probability of being selected for the sex-, grade-, and school type-specific distributions for the region [25, 26]. SPSS (version 21.0; SPSS Inc., Chicago, IL) was used to perform all statistical analyses, and a p-value less than 0.05 was considered to be significant.

## Results

The estimated prevalence of highly frequent energy drink intake (≥5 times a week) was 1.4 %, and prevalence of moderately frequent energy drink intake (1–4 times/week) was 10.5 %. Compared to those who had energy drinks less than once a week, those who had energy drinks 5 times or more per week were more likely to be male (OR = 1.79), attend high school (OR = 1.21), live in a rural area (OR = 1.19), achieve higher academic performance (OR = 1.35), eat junk food (OR = 5.70), drink alcohol (OR = 1.35), be physically active (OR = 1.43), and not to live with their family (OR = 3.73). Those whose energy drink intake frequency was between 1–4 times per week showed similar sociodemographic and lifestyle characteristics as those whose energy drink intake was 5 times or more per week, except that they were less likely to achieve higher academic performance compared to those whose energy drink intake was less than once per week (OR = 0.87) (Table 1).

**Table 1:** Sociodemographic and lifestyle characteristics of adolescents with frequent energy drink and infrequent energy drink consumption

	Highly frequent energy drinks (≥5/wks) (*N* = 945)	Moderately frequent energy drinks (1-4/wks) (*N* = 7262)	Infrequent energy drinks (*N* = 59836)	Highly frequent energy drinks vs. infrequent energy drinks	Moderately frequent energy drinks vs. infrequent energy drinks
	%	%	%	OR (95 % CI)	OR (95 % CI)
Sex, males	65.0	59.8	51.0	1.79 (1.75-1.82)	1.43 (1.42-1.44)
High school	57.5	54.0	52.8	1.21 (1.18-1.23)	1.05 (1.04-1.06)
Rural residence	7.2	8.1	6.1	1.19 (1.14-1.23)	1.36 (1.35-1.38)
Non-residence with family	13.5	5.5	4.0	3.73 (3.63-3.83)	1.40 (1.38-1.43)
High academic achievement	16.4	11.2	12.7	1.35 (1.32-1.38)	0.87 (0.86-0.87)
Junk food consumption (≥3 times /wk)	46.5	23.8	13.2	5.70 (5.59-5.80)	2.05 (2.03-2.07)
Lifetime alcohol use	54.6	49.0	39.6	1.35 (1.34-1.37)	1.46 (1.45-1.48)
Physically active	47.4	42.5	38.7	1.43 (1.40-1.45)	1.17 (1.16-1.18)
	Mean (SD)	Mean (SD)	Mean (SD)	Mean difference (95 % CI)	Mean difference (95 % CI)
Age, yrs	15.44 (1.78)	15.13 (1.76)	15.07 (1.72)	0.37 (0.35-0.39)	0.06 (0.05-0.07)

After adjusting for age, gender, school type, area of residence, residential type, academic achievement, junk food and alcohol consumption, and physical activity, participants who used energy drinks frequently were more likely to experience sleep dissatisfaction (adjusted odd ratios [AORs] = 1.64 and 1.25), severe stress (AORs = 2.23 and 1.38), depressive mood (AOR = 2.59 and 1.51), suicidal ideation (AORs = 3.14 and 1.43), suicide plan (AORs = 4.65 and 1.78), and suicide attempt (AORs = 6.79 and 1.91), compared to participants who used energy drinks infrequently or did not use energy drinks, with higher AORs for highly frequent use of energy drinks than for moderately frequent use of energy drinks (Table 2).

**Table 2:** Association between energy drink intake and mental health problems in Korean adolescents

	Highly frequent energy drinks (≥5/wks) (*N* = 945)	Moderately frequent energy drinks (1-4/wks) (*N* = 7262)	Infrequent energy drinks (*N* = 59836)	Highly frequent energy drinks vs. infrequent energy drinks	Moderately frequent energy drinks vs. infrequent energy drinks
	%	%	%	AOR (95 % CI)	AOR (95 % CI)
Sleep dissatisfaction	52.0	43.2	37.8	1.64 (1.61-1.67)	1.25 (1.25-1.26)
Perceived stress	53.1	41.7	34.3	2.23 (2.19-2.27)	1.38 (1.37-1.39)
Persistent depressive mood	46.0	31.0	22.3	2.59 (2.54-2.65)	1.51 (1.49-1.52)
Suicidal ideation	29.9	15.5	10.9	3.14 (3.07-3.21)	1.43 (1.42-1.45)
Suicide plan	18.2	6.4	3.3	4.65 (4.53-4.78)	1.78 (1.75-1.81)
Suicide attempt	15.1	4.2	2.0	6.79 (6.59-7.00)	1.91 (1.87-1.95)

AOR: odd ratios adjusted for age, gender, school type, residential area, residence type, academic achievement, alcohol and junk food consumption, and physical activity

In order to further examine the nature of the relationships observed, the frequency of energy drink intake and junk food consumption variables were combined, and the prevalence of mental health problems across the four groups according to frequent/infrequent intake were compared. Compared to the infrequent energy drinks and infrequent junk food group, the frequent energy drinks and/or frequent junk food group were more likely to experience sleep dissatisfaction (AORs = 1.58, 1.32, and 1.30), severe stress (AORs = 1.78, 1.47, and 1.24), depressed mood (AORs = 2.48, 1.55, and 1.39), suicidal ideation (AORs = 2.55, 1.47, and 1.30), suicide plan (AORs = 3.94, 2.96, and 1.65), and suicide attempt (AORs = 5.01, 2.11, and 1.58), with the highest AORs for the frequent energy drinks and frequent junk food group followed by the frequent energy drinks and infrequent junk food group and the infrequent energy drinks and frequent junk food group (Table 3). In particular, the frequent energy drinks and frequent junk food group showed significantly higher risk for depressive mood, suicidal ideation, suicide plan, and suicide attempt compared to the other three groups.

**Table 3:** Comparisons of mental health problems according to energy drink and junk food consumption

	Frequent energy drinks/frequent junk food (A) (*N* = 2123)	Frequent energy drinks/infrequent junk food (B) (*N* = 6084)	Infrequent energy drinks/frequent junk food (C) (*N* = 7746)	Infrequent energy drinks/infrequent junk food (D) (*N* = 52090)	C versus D	B versus D	A versus D
	%	%	%	%	AOR (95 % CI)	AOR (95 % CI)	AOR (95 % CI)
Sleep dissatisfaction	47.9	43.0	43.7	36.9	1.30 (1.29-1.31)	1.32 (1.31-1.33)	1.58 (1.56-1. 60)
Perceived stress	45.6	42.2	38.8	33.7	1.24 (1.23-1.25)	1.47 (1.46-1.48)	1.78 (1.76-1.80)
Persistent depressive mood	41.7	29.6	28.2	21.4	1.39 (1.38-1.40)	1.55 (1.54-1.56)	2.48 (2.45-2.51)
Suicidal ideation	24.0	14.7	13.7	10.5	1.30 (1.29-1.32)	1.47 (1.45-1.49)	2.55 (2.51-2.59)
Suicide plan	13.2	5.9	5.1	3.0	1.65 (1.62-1.68)	1.96 (1.93-1.99)	3.94 (3.86-4.03)
Suicide attempt	10.0	3.9	3.1	1.9	1.58 (1.54-1.61)	2.11 (2.07-2.16)	5.01 (4.89-5.14)

AOR: odd ratios adjusted for age, gender, school type, area of residence, residential type, level of academic achievement, level of physical activity, and alcohol use

## Discussion

Our findings indicate that energy drink consumption is associated with multiple mental health problems in adolescents. Our findings also reveal this deleterious effect was most significant in combination with junk food consumption.

In the present study, approximately 12 % of adolescents consumed energy drinks at least once every week. The adolescents with frequent energy drink intake were more likely to be male and physically active, which is consistent with previous findings [28, 29]. Masculinity and sports-related identity may be associated with male adolescents’ frequent energy drink consumption [29]. The adolescents with frequent energy drink intake, in particular those who consumed energy drinks 5 or more days per week, also tended to reside in a rural area where access to healthy food are limited [30]. It is possible that being separated from family may affect their unregulated dietary habits [31, 32]. In addition they were inclined to consume junk food, which suggests that energy drink and junk food consumption habits tend to co-occur. Our results are compatible with previous findings indicating that junk food consumption is accompanied by other unhealthy eating behaviors [21, 33]. It is notable that highly frequent energy drink consumption, but not moderately frequent consumption, was positively related to high academic achievement in our study. This finding was somewhat inconsistent with previous studies that reported the association between energy drink intake and lower academic performance [28, 34]. Considering the tendency to increase study hours at the cost of sleeping hours among Korean students [8], students whose grades are in the upper ranks may habitually use highly caffeinated drinks with high frequency for its wakening effect. Although some students may have benefited from the instant physiological effects to enhance cognitive performances [35], further research is needed to clarify the effect of energy drink intake on students’ academic performance in the long term.

Frequent energy drink intake was significantly associated with a wide range of mental health problems, including sleep dissatisfaction, stress, depressive mood, and suicidality, irrespective of sociodemographic factors, physical activity, alcohol use, and junk food consumption. Particularly, adolescents who consumed energy drinks for 5 days or more every week were at the greatest risk of the aforementioned mental health problems. These findings are consistent with previous studies on the adverse health effects of energy drink consumption [5, 34, 36–38]. In particular, considering a relatively high prevalence of suicidal behavior (i.e., suicidal ideation rates 19.1 % and suicide attempt rates 4.9 %) in Korean adolescents [39, 40], it should be noted that suicide attempt in adolescents who consumed energy drinks five times or more per week was 7 times as high as those adolescents who consumed energy drinks less than once per week.

Caffeine, the main active ingredient in energy drinks, reduces both sleep duration and sleep quality, manifested by increased wake time after sleep onset and decreased proportion of deep sleep [15, 41, 42]. This may lead to subsequent consumption in reaction to feeling tired on the following day, which can create a vicious cycle of energy drink consumption and poor sleep quality. Also, caffeine overdose can induce nervousness and irritable mood [4], and abstinence of caffeine is highly associated with dysphoric mood states for habitual consumers, which implies caffeine withdrawal [43, 44]. The mood fluctuations and irritability caused by caffeine overdose or withdrawal may account for high levels of perceived stress, depressive mood symptoms, and suicidal behavior in the adolescents who frequently consumed energy drinks.

Taken together, although energy drink consumption may have temporary psychoactive effects such as mood enhancement or reduced fatigue [45, 46], the current study sheds more light on its potential detrimental effects. There is an argument that the removal of the negative consequences of caffeine withdrawal, such as drowsiness or dysphoric mood, may account for the seemingly positive effects of caffeine [47–49]. Accordingly, adolescents who consume energy drinks may frequently experience decreased alertness and depressive mood as withdrawal symptoms of caffeine, which may cause dependence on the caffeinated beverages in order to maintain the normal level of activation, rather than benefitting from its positive effects.

As hypothesized, energy drink intake combined with frequent junk food consumption showed the highest risk for mental health problems in general. Food additives, sugar intake, a low micro-nutrient intake, and polyunsaturated fatty acid (PUFA) deficiency associated with frequent junk food consumption predisposes children to behavioral symptoms, such as hyperactivity, delinquency, and aggressive behaviors [50–53]. In prior studies, chronic high fat food consumption led to hypo-affective states with increased hypothalamic-pituitary-adrenocortical axis reactivity [54], and changed brain reward circuitry into depressive behaviors with an increased brain-derived neurotrophic factor (BDNF) and cAMP response element binding protein (CREB) activity [55]. In addition, consumption of sweetened desserts was reported to lead to peaks and troughs in blood sugar, along with associated periods of hyperactivity and lethargy [20, 52]. Thus, frequent junk food consumption can aggravate the mood effects of caffeine contained in energy drinks. Considering the aforementioned association between junk food consumption and energy drink intake, the danger of adolescents’ associated dietary habits demands special attention.

This study has several limitations that deserve discussion. First, due to the cross-sectional design of this study, causal relationships between energy drink intake and mental health problems cannot be determined. Given that energy drink consumption did not predict psychiatric problems at 6-month follow up [38], the association may be bi-directional and reverse causality should also be considered. For example, adolescents who are under stress may be more likely to consume energy drinks for an instant mood elevation effect [56]. Second, sleep dissatisfaction, perceived stress, depressive mood, and suicidality were measured using one item each, rather than with standardized multi-item measures. This may limit the validity of measurements of these variables [26]. Third, information on the constituents of energy drinks and the type of junk food subjects were lacking. Energy drinks contain various ingredients such as caffeine, sugar, guarana, taurine, ginseng, and bitter orange [10]. There are several evidences supporting the differential effects of these ingredients on cognitive performance and mood [35, 57, 58], although only a few studies investigated the interactive cognitive effects of caffeine, taurine and sugar [35, 57]. Further researches are needed to clarify the interactive effects of various ingredients of energy drinks on mood and behaviors. Fourth, data about the number of years the subjects were consuming the drink or the total amount of energy drink intake were also lacking. Finally, energy drink intake and suicide attempts may have been underreported because of concerns over disclosing suicide attempts and frequent use of energy drinks. Therefore, future studies are needed to further clarify the causal relationships among energy drink intake, emotional problems, and other associated food consumption. However, the strength of the present study is its inclusion of data from 68,043 adolescents from a nationally representative sample in South Korea, which improves the external validity of the study results.

## Conclusions

In conclusion, study findings indicate that adolescents who consume energy drinks regularly are at great risk of mental health problems, particularly when in combination with junk food consumption. Furthermore, the risk of mental health problems was significantly different between highly frequent and moderately frequent consumers. Therefore, the amount of energy drink intake should be carefully monitored, and an educational intervention on the negative effects of energy drinks is needed for adolescents who consume energy drinks regularly.

## Abbreviations

BDNF: Brain-derived neurotrophic factor
CIs: Confidence intervals
CREB: cAMP response element binding protein
KYRBS: Korean Youth Risk Behavior Web-Based Survey
OECD: Organization for Economic Cooperation & Development
ORs: Odds ratios
PUFA: Polyunsaturated fatty acid
